# Multidrug Resistance, Antimicrobial Therapy, and In-Hospital Mortality in Patients with Healthcare-Associated Infections: A Retrospective Cohort Study

**DOI:** 10.3390/antibiotics15101009

**Published:** 2026-10-11

**Authors:** Septimiu Voidazan, Réka Bodea, Liviu Moraru, Iuliu Moldovan, Daniela Iancu, Mihaela Alexandra Budianu

**Affiliations:** 1 Department of Epidemiology, University of Medicine, Pharmacy, Science and Technology George Emil Palade of Targu-Mures, 540141 Targu Mures, Romania; septimiu.voidazan@umfst.ro (S.V.); mihaela.budianu@umfst.ro (M.A.B.); 2 Department of Anatomy, University of Medicine, Pharmacy, Science and Technology George Emil Palade of Targu-Mures, 540139 Targu Mures, Romania; liviu.moraru@umfst.ro; 3 Department of Public Health and Health Management, University of Medicine, Pharmacy, Science and Technology George Emil Palade of Targu-Mures, 540141 Targu Mures, Romania; iuliu.moldovan@umfst.ro; 4 Doctoral School, University of Medicine, Pharmacy, Science and Technology George Emil Palade of Targu-Mures, 540141 Targu Mures, Romania; daniela.iancu@umfst.ro

**Keywords:** healthcare-associated infections, antimicrobial resistance, multidrug resistance, MDR, in-hospital mortality, antimicrobial therapy, *Acinetobacter baumannii*, antimicrobial stewardship

## Abstract

**Background:** Healthcare-associated infections (HAIs), particularly those caused by antimicrobial-resistant pathogens, remain an important cause of adverse outcomes among hospitalized patients. The combined contribution of antimicrobial resistance, clinical characteristics, and antimicrobial treatment to mortality requires further evaluation. This study aimed to investigate factors associated with in-hospital mortality among patients with HAIs, with particular emphasis on multidrug resistance (MDR) and post-culture antimicrobial treatment. **Methods:** We conducted a retrospective observational cohort study that included 192 patients with 242 documented HAI episodes in a hospital in Târgu-Mureș, Romania. Demographic and clinical characteristics, invasive procedures, microbiological findings, antimicrobial resistance phenotypes, antimicrobial exposure, and in-hospital outcome were evaluated. Comparisons between survivors and non-survivors were performed using the Mann–Whitney U test, Pearson’s chi-square test, or Fisher’s exact test, as appropriate. Factors associated with in-hospital mortality were investigated using univariable and multivariable logistic regression. **Results:** Overall, 161/192 patients (83.9%) died during hospitalization. Non-survivors were significantly older than survivors [median: 71 (IQR: 62–78) vs. 62 (50.5–72) years; *p* = 0.025]. MDR organisms were more common among non-survivors (51.6% vs. 22.6%; *p* = 0.003), and MDR phenotype was associated with increased odds of mortality in univariable analysis (OR = 3.65, 95% CI: 1.49–8.95; *p* = 0.005). After adjustment for age, mechanical ventilation, and recent surgery, MDR remained independently associated with in-hospital mortality (aOR = 3.58, 95% CI: 1.42–8.98; *p* = 0.007). In the treatment-focused model, aminopenicillin exposure (aOR = 0.21, 95% CI: 0.07–0.64; *p* = 0.006), second-generation aminoglycosides (aOR = 0.23, 95% CI: 0.06–0.97; *p* = 0.045), and third-generation cephalosporins (aOR = 0.35, 95% CI: 0.14–0.88; *p* = 0.025) were associated with lower odds of mortality. No significant association was observed between time to specific antimicrobial therapy and mortality (*p* = 0.541). **Conclusions:** MDR was the main antimicrobial-resistance factor independently associated with in-hospital mortality among patients with HAIs. Although several antimicrobial classes were associated with lower odds of death, these observational associations should not be interpreted as causal treatment effects. The findings emphasize the importance of antimicrobial resistance surveillance, rapid microbiological diagnosis, antimicrobial stewardship, and timely access to active antimicrobial therapy.

## 1. Introduction

Healthcare-associated infections (HAIs) remain a major patient-safety challenge and contribute substantially to morbidity, mortality, prolonged hospitalization, antimicrobial consumption, and healthcare expenditure. The World Health Organization (WHO) emphasizes that HAIs continue to affect patients and healthcare systems worldwide and represent an important driver of antimicrobial resistance (AMR), while a substantial proportion of these infections could be prevented through effective infection prevention and control measures [1].

In Europe, the most recent point-prevalence survey conducted by the European Centre for Disease Prevention and Control (ECDC) included more than 290,000 patients from 1250 acute-care hospitals in the EU/EEA, confirming the continuing substantial burden of HAIs and antimicrobial use across European healthcare systems [2]. Earlier European burden analyses have similarly demonstrated that HAIs account for a considerable loss of healthy life-years and represent a major component of the overall burden of communicable diseases [3].

The clinical impact of HAIs is increasingly compounded by antimicrobial resistance. Globally, bacterial AMR is associated with a substantial mortality burden, and large-scale analyses have identified resistant bacterial infections as an important and growing threat to population health [4]. Of particular concern in healthcare settings are multidrug-resistant (MDR), extensively drug-resistant (XDR), and pandrug-resistant (PDR) organisms, which restrict therapeutic options and may increase the likelihood of initially ineffective antimicrobial treatment. The WHO Bacterial Priority Pathogens List 2024 highlights several resistant Gram-negative pathogens of major public-health importance, including carbapenem-resistant *Acinetobacter baumannii*, carbapenem-resistant *Pseudomonas aeruginosa*, and resistant *Enterobacterales* [5]. These microorganisms are particularly relevant in critically ill patients, who frequently experience prolonged hospitalization, invasive procedures, mechanical ventilation, central venous catheterization, and extensive previous antimicrobial exposure.

Among these pathogens, *Acinetobacter baumannii* represents a major challenge because of its ability to persist in healthcare environments and acquire resistance to multiple antimicrobial classes. High levels of multidrug resistance have been reported among *A. baumannii* isolates responsible for hospital-acquired and ventilator-associated pneumonia, with substantial associated mortality [6]. Carbapenem resistance further complicates the management of *A. baumannii* infections and has been associated with adverse clinical outcomes [7]. Nevertheless, the relationship between antimicrobial resistance and mortality is complex. Mortality among patients infected with resistant organisms may reflect not only the resistance phenotype itself but also advanced age, underlying disease, severity of illness, invasive procedures, infection site, and delays in receiving active antimicrobial therapy. Indeed, previous cohort studies have produced heterogeneous findings regarding whether multidrug resistance itself remains independently associated with mortality after adjustment for these clinical factors [8].

Antimicrobial treatment represents another key determinant of clinical outcome. The therapeutic management of HAIs caused by resistant organisms requires a balance between sufficiently broad empirical coverage and antimicrobial stewardship. Inappropriate empirical therapy is more likely when infections are caused by resistant microorganisms and may adversely affect survival. A large cohort study of more than 21,000 patients with bloodstream infections demonstrated that discordant empirical antimicrobial therapy was independently associated with increased mortality, while antimicrobial resistance strongly predicted the receipt of discordant treatment [9]. Similarly, a systematic review and meta-analysis including 198 studies and nearly 90,000 patients with bacteremia found that inappropriate empirical antimicrobial therapy was associated with approximately twice the odds of mortality compared with appropriate therapy [10]. More recent studies have also reported lower mortality among patients receiving antimicrobial treatment that is active against the causative Gram-negative pathogen [11,12].

However, evaluating the relationship between individual antimicrobial agents and mortality in observational studies remains challenging. Antimicrobial selection is influenced by infection severity, previous antibiotic exposure, pathogen identity, susceptibility profile, organ dysfunction, and availability of therapeutic alternatives. Consequently, associations between specific antimicrobial classes and clinical outcomes may be affected by confounding by indication and should not automatically be interpreted as causal treatment effects. An integrated assessment incorporating patient characteristics, invasive procedures, microbiological findings, resistance phenotypes, and antimicrobial exposure is therefore necessary to better characterize the determinants of mortality among patients with HAIs.

Therefore, this study aimed to investigate the clinical, microbiological, antimicrobial-resistance, and treatment-related factors associated with in-hospital mortality among patients with HAIs. Particular emphasis was placed on determining whether MDR phenotype was independently associated with mortality after adjustment for relevant clinical factors and on evaluating the relationship between post-culture antimicrobial treatment and patient outcome. We hypothesized that MDR infection would be independently associated with increased in-hospital mortality and that antimicrobial treatment patterns would differ between survivors and non-survivors.

## 2. Results

The study cohort comprised patients initially hospitalized in various clinical and surgical departments who were subsequently transferred to the Intensive Care Unit (ICU). The largest recorded source departments were Infectious Diseases (47/192; 24.5%), Pneumology (25/192; 13.0%), Psychiatry (16/192; 8.3%), General Surgery (13/192; 6.8%), Cardiology (13/192; 6.8%), and the surgical operating block (12/192; 6.3%); additional patients came from Internal Medicine, Gastroenterology, Neurology, Orthopedics, Plastic Surgery, and emergency-department/ward combinations.

**Figure 1:**
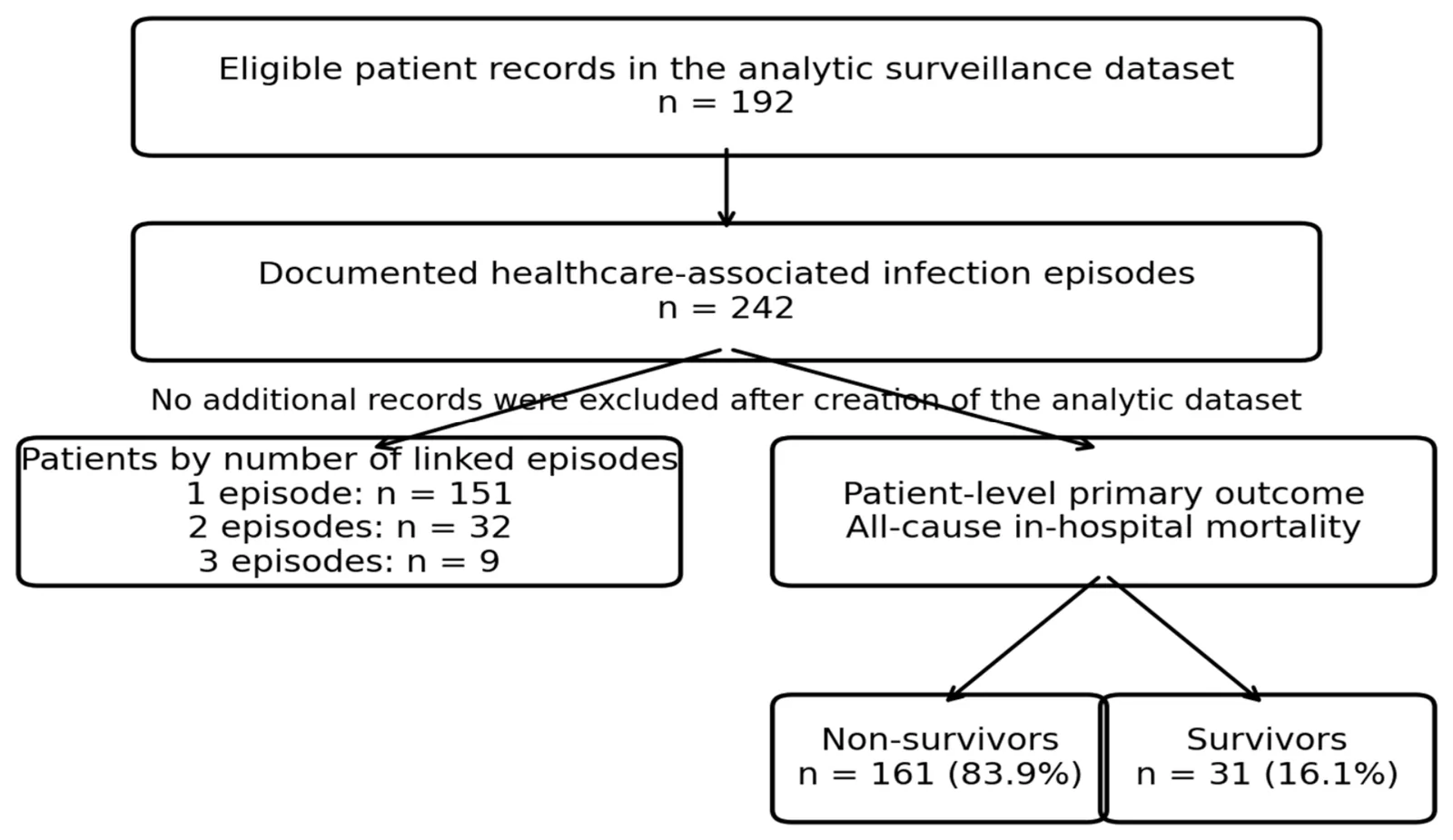
Patient-selection and episode-structure flow diagram.

Microbiological specimens were predominantly respiratory: 129/242 (53.3%) tracheal aspirates, 10/242 (4.1%) sputum samples, and 9/242 (3.7%) bronchial aspirates. Other specimens included 36/242 (14.9%) blood-culture samples, 39/242 (16.1%) purulent secretions, 14/242 (5.8%) urine cultures, 4/242 (1.7%) central venous catheter samples, and 1/242 (0.4%) conjunctival secretion (Figure 1). The surveillance extract did not contain a sufficiently standardized anatomical HAI-site variable to retrospectively classify all episodes as pneumonia, bloodstream infection, UTI, or surgical-site infection without introducing post hoc misclassification; specimen type is therefore reported separately.

Among the 192 patients included in the study, 161 (83.9%) died during hospitalization and 31 (16.1%) survived. Non-survivors were significantly older than survivors, with a median age of 71 years (IQR: 62–78) versus 62 years (IQR: 50.5–72), respectively (*p* = 0.025). Recent surgery was more frequently documented among survivors than among non-survivors (35.5% vs. 17.4%, *p* = 0.022). Mechanical ventilation was highly prevalent in both groups and was more frequently administered to non-survivors (91.9% vs. 80.6%), although the difference did not reach statistical significance (*p* = 0.092). No significant differences according to in-hospital outcome were observed for sex, place of residence, cardiovascular, pulmonary, oncological, metabolic or renal comorbidities, central venous catheterization, previous antibiotic exposure, or length of hospital stay (Table 1).

Marked differences in antimicrobial resistance patterns were observed according to in-hospital outcome. MDR organisms were significantly more common among non-survivors than among survivors (51.6% vs. 22.6%, *p* = 0.003). Carbapenem-resistant (CR) phenotypes were recorded in 55 patients and were more common among survivors (45.2%) than non-survivors (25.5%; *p* = 0.026), corresponding to a crude OR below 1. This finding should not be interpreted as a protective effect of carbapenem resistance. In the source coding, MDR and CR were separate phenotype indicators: 90 patients were classified as MDR without CR, 55 as CR without MDR, and 47 as neither; no patient was simultaneously coded as MDR and CR (Table 2). Among CR-coded patients, selected organism-specific indicators included carbapenem-resistant *A. baumannii* in 15 patients, carbapenem-resistant *P. aeruginosa* in 5, and CPE-producing *K. pneumoniae* in 11. Polymyxins were administered to 28/55 (50.9%) CR-coded patients compared with 50/137 (36.5%) patients without CR. These descriptive findings support confounding by organism, phenotype definition, treatment selection, and case mix as plausible explanations for the inverse crude CR association. XDR status was not significantly associated with mortality. MDR *A. baumannii* was substantially more common among non-survivors (47.8% vs. 12.9%, *p* < 0.001). *A. baumannii* CR alone did not differ significantly between outcome groups (*p* = 0.072), (Table 3).

**Figure 2:**
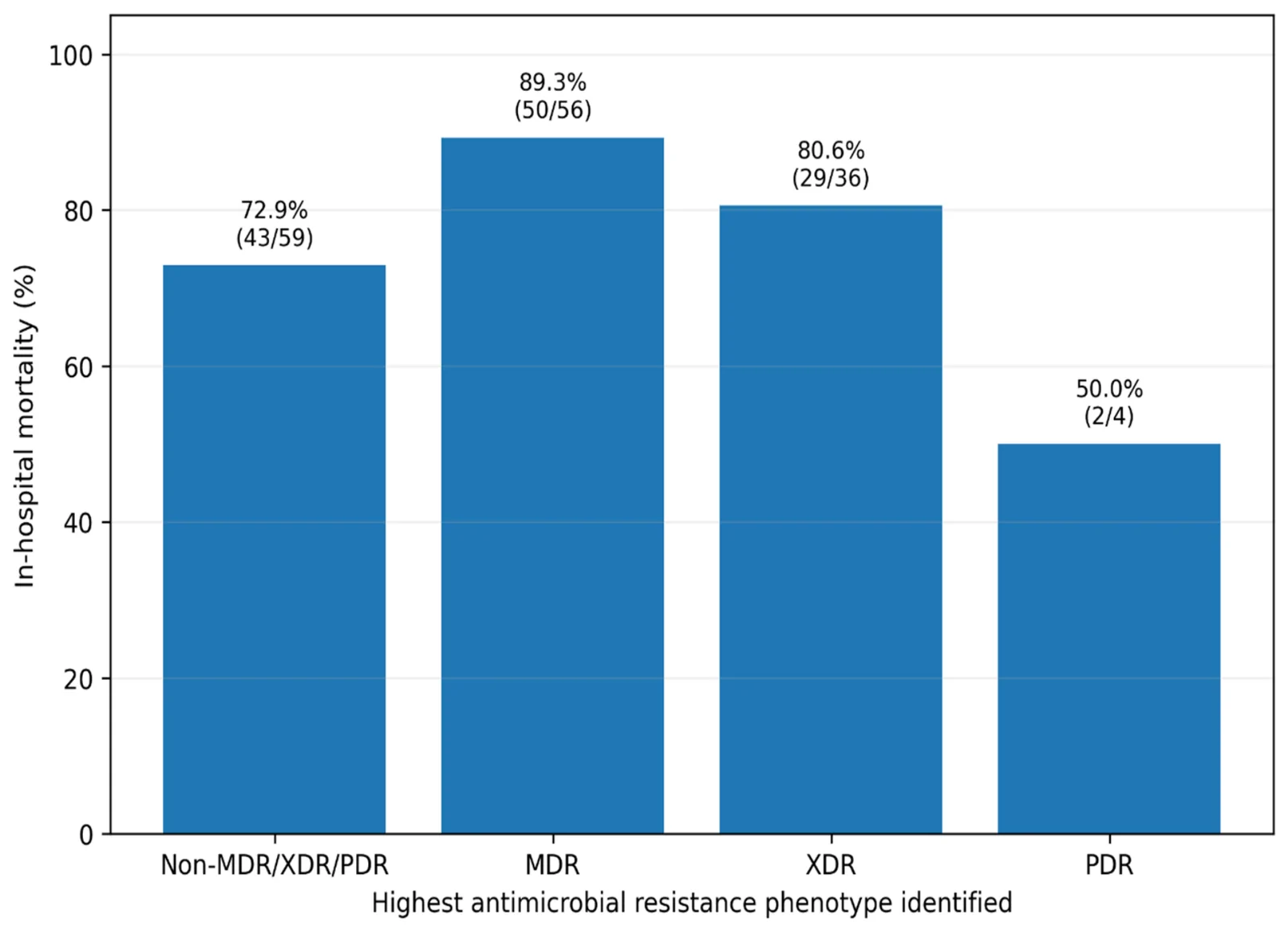
In-hospital mortality according to antimicrobial resistance phenotype. Patients were classified according to the highest antimicrobial resistance phenotype identified during hospitalization. In-hospital mortality was 72.9% (43/59) among patients without an MDR/XDR/PDR phenotype, 89.3% (50/56) among patients with MDR organisms, 80.6% (29/36) among those with XDR organisms, and 50.0% (2/4) among patients with PDR organisms.

When patients were classified according to the highest antimicrobial resistance phenotype identified during hospitalization, in-hospital mortality was highest among patients with MDR organisms (89.3%; 50/56), compared with 80.6% (29/36) among patients with XDR organisms and 72.9% (43/59) among those without an MDR/XDR/PDR phenotype (Figure 2). Mortality was 50.0% (2/4) in the PDR subgroup; however, this estimate should be interpreted cautiously because of the very small number of patients in this category.

Post-culture antimicrobial treatment patterns differed between survivors and non-survivors. Aminopenicillins were administered more frequently among survivors than non-survivors (32.3% vs. 6.8%, *p* < 0.001), as were second-generation aminoglycosides (22.6% vs. 3.1%, *p* < 0.001) and third-generation cephalosporins (54.8% vs. 31.1%, *p* = 0.011). No statistically significant differences were observed for fluoroquinolones, glycylcyclines, oxazolidinones, glycopeptides, carbapenems, or polymyxins.

The median time from microbiological sampling to initiation of specific antimicrobial therapy was 6 days in both non-survivors and survivors and did not differ significantly between the groups (*p* = 0.541). In contrast, the median duration of antibiotic treatment was significantly shorter among non-survivors than survivors [4 days (IQR: 2–8) vs. 9 days (IQR: 5–19), *p* < 0.001], (Table 4).

**Figure 3:**
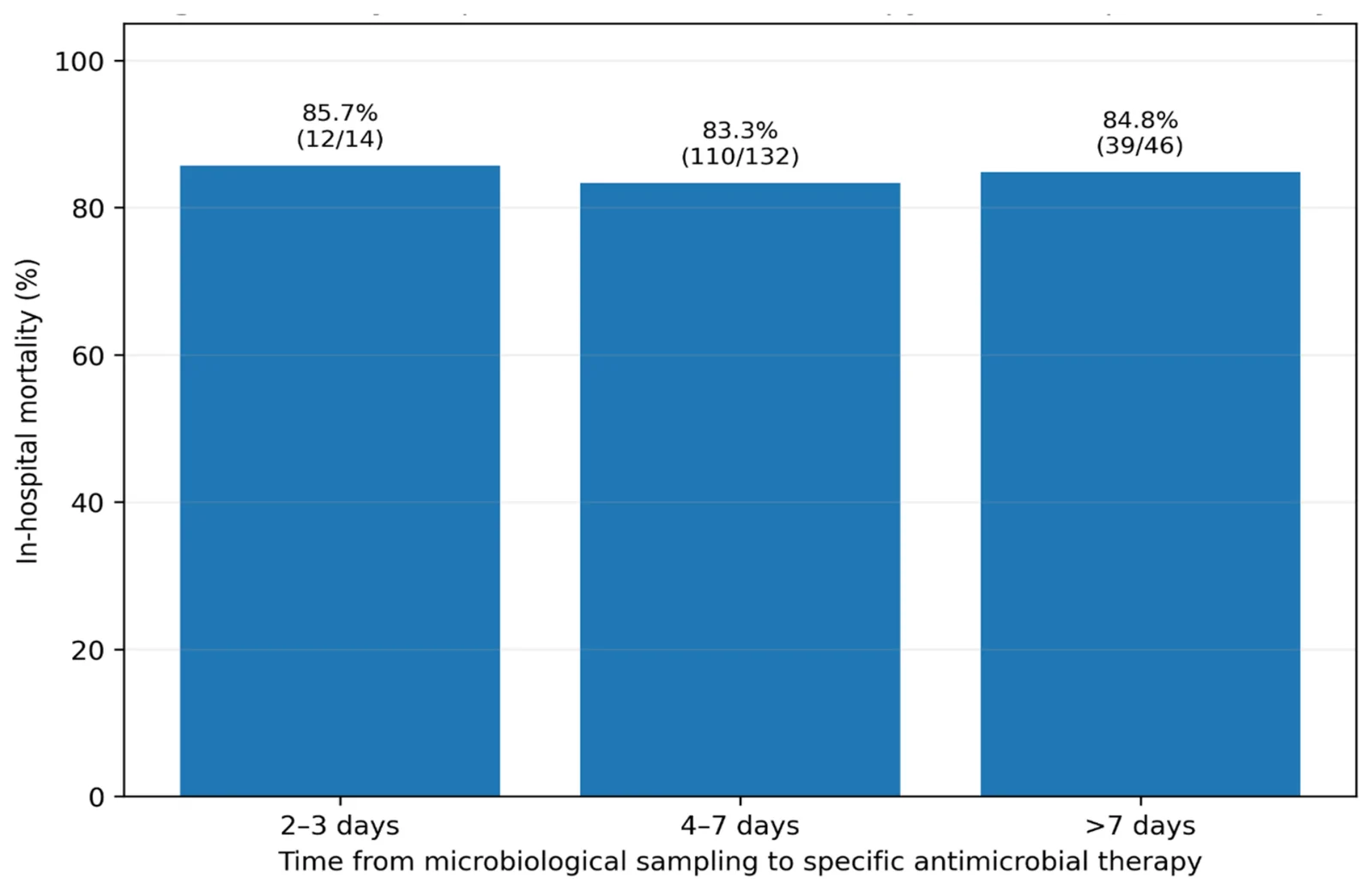
Delay until start of specific antimicrobial therapy and in-hospital mortality.

No clear relationship was observed between increasing time to initiation of recorded post-culture antimicrobial therapy and in-hospital mortality (Figure 3). Mortality was 85.7% (12/14) among patients treated within 2–3 days, 83.3% (110/132) among those treated within 4–7 days, and 84.8% (39/46) among those treated after >7 days. These findings were consistent with the continuous analysis, in which the median time to recorded post-culture therapy was 6 days in both survivors and non-survivors (*p* = 0.541). The treatment was not necessarily microbiologically active because drug-isolate susceptibility matching was unavailable.

In univariable logistic regression analysis, increasing age was associated with higher odds of in-hospital mortality (OR per 1-year increase = 1.03, 95% CI: 1.00–1.06; *p* = 0.031). The presence of an MDR phenotype was strongly associated with mortality, corresponding to more than a threefold increase in the odds of death (OR = 3.65, 95% CI: 1.49–8.95; *p* = 0.005). Mechanical ventilation was also associated with higher odds of mortality (OR = 2.73, 95% CI: 0.95–7.86), although the association did not reach statistical significance (*p* = 0.062). Recent surgery was inversely associated with mortality (OR = 0.38, 95% CI: 0.17–0.89; *p* = 0.025).

Regarding antimicrobial treatment, exposure to aminopenicillins (OR = 0.15, 95% CI: 0.06–0.41; *p* < 0.001), second-generation aminoglycosides (OR = 0.11, 95% CI: 0.03–0.37; *p* < 0.001), third-generation aminoglycosides (OR = 0.31, 95% CI: 0.10–0.99; *p* = 0.048), and third-generation cephalosporins (OR = 0.37, 95% CI: 0.17–0.81; *p* = 0.013) was associated with lower odds of in-hospital mortality in univariable analysis. Carbapenem and polymyxin exposure was not significantly associated with mortality (Table 5).

In the clinical and antimicrobial-resistance multivariable model, MDR phenotype remained independently associated with in-hospital mortality after adjustment for age, mechanical ventilation, and recent surgery (aOR = 3.58, 95% CI: 1.42–8.98; *p* = 0.007). Age showed a borderline association with mortality (aOR per 1-year increase = 1.03, 95% CI: 1.00–1.06; *p* = 0.067). Mechanical ventilation was associated with an approximately 2.8-fold increase in the odds of death, although the association did not reach statistical significance (aOR = 2.79, 95% CI: 0.88–8.88; *p* = 0.082). Recent surgery was no longer significantly associated with mortality after multivariable adjustment (aOR = 0.48, 95% CI: 0.20–1.19; *p* = 0.113).

In the treatment-focused multivariable model, aminopenicillin exposure remained associated with lower odds of in-hospital mortality (aOR = 0.21, 95% CI: 0.07–0.64; *p* = 0.006). Similar independent associations were observed for second-generation aminoglycosides (aOR = 0.23, 95% CI: 0.06–0.97; *p* = 0.045) and third-generation cephalosporins (aOR = 0.35, 95% CI: 0.14–0.88; *p* = 0.025), after adjustment for age, mechanical ventilation, and recent surgery (Table 6).

No independent association with mortality was observed for the remaining clinical covariates included in the treatment-focused model.

**Figure 4:**
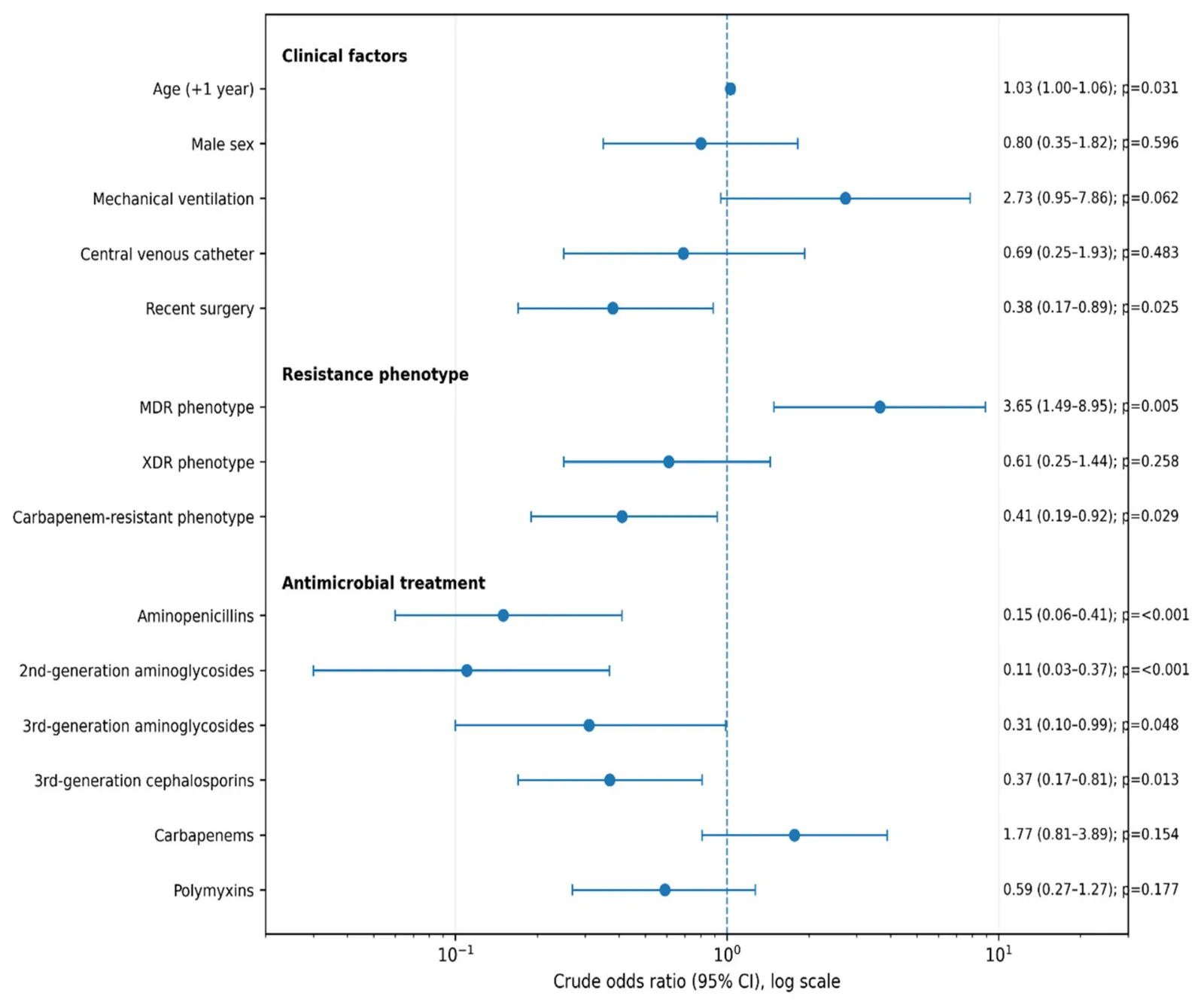
Forest plot of univariable associations between clinical characteristics, antimicrobial resistance phenotypes, antimicrobial exposure, and in-hospital mortality. Odds ratios (ORs) and 95% confidence intervals (CIs) are shown on a logarithmic scale. The vertical dashed line indicates the null value (OR = 1).

Figure 4 summarizes the magnitude and direction of the associations between clinical characteristics, antimicrobial resistance phenotypes, antimicrobial treatment, and in-hospital mortality. MDR phenotype showed the strongest positive association with mortality (OR = 3.65, 95% CI: 1.49–8.95; *p* = 0.005), while mechanical ventilation was associated with higher odds of death but did not reach statistical significance (OR = 2.73, 95% CI: 0.95–7.86; *p* = 0.062). Several antimicrobial exposures, including aminopenicillins, second- and third-generation aminoglycosides, and third-generation cephalosporins, were associated with significantly lower odds of in-hospital mortality in univariable analysis.

Sensitivity analyses restricted the exposure definition to the index microbiological episode. Index-episode MDR remained associated with in-hospital mortality (81/87 [93.1%] deaths among patients with index-episode MDR vs. 80/105 [76.2%] among those without index-episode MDR; crude OR = 4.22, 95% CI: 1.64–10.83; *p* = 0.003). After adjustment for age, mechanical ventilation, and recent surgery, the association remained significant (aOR = 4.07, 95% CI: 1.55–10.71; *p* = 0.004). In index-episode treatment sensitivity models adjusted for the same clinical covariates, aminopenicillin exposure (aOR = 0.21, 95% CI: 0.07–0.60; *p* = 0.004), second-generation aminoglycoside exposure (aOR = 0.14, 95% CI: 0.04–0.50; *p* = 0.002), and third-generation cephalosporin exposure (aOR = 0.29, 95% CI: 0.12–0.67; *p* = 0.004) remained inversely associated with mortality. These treatment associations remain exploratory and do not resolve confounding by indication or immortal-time bias.

## 3. Discussion

The present study evaluated clinical, microbiological, antimicrobial-resistance, and treatment-related factors associated with in-hospital mortality among 192 patients with 242 healthcare-associated infection episodes. The principal finding was the strong association between multidrug resistance and mortality. MDR organisms were substantially more common among non-survivors than survivors, and MDR phenotype remained independently associated with in-hospital death after adjustment for age, mechanical ventilation, and recent surgery. This finding suggests that, within this cohort, the adverse prognostic significance of multidrug resistance could not be explained solely by the major clinical characteristics included in the adjusted model.

The relationship between antimicrobial resistance and mortality has been consistently recognized as clinically important, although the magnitude of the effect varies substantially according to pathogen, infection site, patient population, and analytical approach.

Global analyses continue to demonstrate a considerable mortality burden attributable to bacterial antimicrobial resistance. More specifically, recent observational evidence has reported significant associations between infection with multidrug-resistant organisms and in-hospital mortality, although residual confounding by severity of illness and comorbidity remains an important consideration [13].

Our finding that MDR infection was associated with more than threefold higher adjusted odds of death is therefore consistent with the broader evidence supporting antimicrobial resistance as an important marker of poor prognosis in hospitalized patients.

The predominance of *Acinetobacter baumannii* and resistant *A. baumannii* phenotypes is also clinically relevant. *A. baumannii* is a major healthcare-associated pathogen, particularly among critically ill and mechanically ventilated patients, and its capacity to accumulate multiple resistance mechanisms severely limits therapeutic options. A systematic review and meta-analysis examining hospital-acquired and ventilator-associated pneumonia caused by *A. baumannii* reported a pooled MDR prevalence of approximately 80% and an overall mortality estimate exceeding 40% [6]. More recent ICU cohorts continue to show particularly poor outcomes in carbapenem-resistant *A. baumannii* infections; for example, a five-year cohort of patients with Gram-negative bacteriemia reported substantially higher overall in-hospital mortality among patients with CRAB bacteraemia than among those infected with other Gram-negative organisms [14]. These findings support the clinical importance of resistant *Acinetobacter* in populations characterized by extensive healthcare exposure and invasive procedures.

Our findings should also be considered in the context of recent Romanian data. Studies conducted in Romanian tertiary-care hospitals have documented a substantial burden of MDR healthcare-associated infections, particularly in intensive care settings, with *Acinetobacter baumannii* consistently emerging as one of the predominant resistant pathogens. However, reported mortality varies considerably across populations and clinical settings. Differences in mortality between our cohort and previous Romanian studies should therefore be interpreted in light of differences in case mix, clinical severity, invasive supportive care, infection characteristics, and antimicrobial-resistance burden [15,16,17].

An important feature of the present cohort was the very high overall in-hospital mortality (83.9%). This figure should not be interpreted as an estimate of HAI-attributable mortality or of mortality among the overall population of patients with HAIs treated at the institution. Rather, it represents all-cause in-hospital mortality within the selected study cohort, which was characterized by a particularly high-risk clinical profile. Importantly, the study cohort consisted of patients transferred from various clinical and surgical departments to the ICU, further indicating that the study population represented a selected group of critically ill hospitalized patients rather than an unselected population of patients with HAIs. More than 90% of patients required mechanical ventilation, approximately four-fifths had a central venous catheter, more than 94% had previous antimicrobial exposure, and some patients experienced multiple HAI episodes during hospitalization. These characteristics reflect substantial healthcare exposure and a critically ill population with a high baseline risk of death. Accordingly, the high observed mortality likely reflects the combined effects of underlying disease severity, invasive supportive care, recurrent or multiple infectious episodes, and antimicrobial resistance, rather than the attributable effect of HAI alone. Mechanical ventilation was more frequently administered to non-survivors and was associated with approximately 2.7-fold higher odds of mortality in univariable analysis; the magnitude of this association remained substantial after multivariable adjustment, although it did not reach conventional statistical significance. This result should not be interpreted as evidence that mechanical ventilation itself causes mortality. Rather, mechanical ventilation is likely to act as a marker of underlying disease severity, critical illness, prolonged exposure to invasive devices, and increased susceptibility to respiratory HAIs. The wide confidence intervals observed in the adjusted model also indicate limited precision, most likely reflecting the relatively small number of survivors.

Age was another clinically relevant factor. Non-survivors were significantly older than survivors, and increasing age was associated with greater odds of mortality in the univariable analysis. After adjustment, the association became borderline and no longer met the conventional threshold for statistical significance. This attenuation suggests that part of the effect of age may overlap with other clinical characteristics and the burden of resistant infection. Nevertheless, the direction of the association is biologically plausible given the reduced physiological reserve, greater burden of chronic disease, and vulnerability to severe infection typically observed in older hospitalized populations.

A second major finding concerned antimicrobial treatment. Exposure to aminopenicillins, second-generation aminoglycosides, and third-generation cephalosporins was associated with lower odds of in-hospital mortality in both the univariable and treatment-focused multivariable analyses. These findings require particularly cautious interpretation. They should not be regarded as evidence that these antimicrobial classes exert an independent protective effect. In observational studies, antimicrobial selection is strongly determined by pathogen susceptibility, infection severity, prior antimicrobial exposure, organ function, and the availability of active therapeutic alternatives. Patients infected with susceptible organisms and with less severe disease are more likely to remain eligible for narrower or conventional antimicrobial regimens, whereas critically ill patients infected with highly resistant organisms are more likely to receive broad-spectrum or last-line agents. Therefore, the observed associations may at least partly reflect confounding by indication and differences in underlying microbiological susceptibility rather than direct treatment effects.

This interpretation is supported by existing evidence showing that the adequacy of antimicrobial therapy is often more prognostically relevant than the antimicrobial class itself.

In a large US cohort of more than 20,000 bloodstream infection episodes, discordant empirical antimicrobial therapy was closely associated with antimicrobial resistance and with increased mortality [9]. Similarly, a systematic review and meta-analysis found that inappropriate empirical antimicrobial therapy was associated with unfavorable mortality outcomes across bacteriemic populations [10]. More recent real-world evidence has likewise shown higher mortality among patients receiving initially inadequate treatment, particularly when resistant bacteria were involved [18]. Consequently, future analyses of this cohort would benefit from direct matching of each administered antimicrobial agent with the susceptibility profile of the corresponding isolate, allowing for the classification of therapy as microbiologically appropriate or inappropriate.

Interestingly, the interval between microbiological sampling and initiation of specific antimicrobial therapy was not significantly associated with mortality in the present study. Mortality remained high and relatively similar across the 2–3-day, 4–7-day, and >7-day categories, and the median interval to specific treatment was 6 days in both survivors and non-survivors. This finding should not be interpreted as evidence that treatment delay is clinically irrelevant. Rather, the variable available in the database represented time to specific therapy, not necessarily time to the first microbiologically active therapy. Studies evaluating sepsis and bloodstream infection populations have reported associations between delayed or inappropriate antimicrobial treatment and poorer outcomes, although the magnitude of the effect varies according to disease severity, infection source, and the definition of treatment adequacy [19]. The absence of a clear association in our cohort may therefore reflect limitations in treatment-timing measurement, heterogeneity of infection episodes, and inability to determine whether the initiated therapy was active against the causative pathogen.

The significantly longer duration of antimicrobial treatment among survivors should also be interpreted cautiously. A longer treatment course cannot be considered protective on the basis of these data because patients who die early inherently have less opportunity to accumulate treatment days. This introduces a form of survivorship or time-dependent bias. Accordingly, treatment duration was retained as a descriptive variable rather than interpreted as a causal determinant of survival.

The findings concerning carbapenem-resistant phenotypes also require a nuanced interpretation. Although carbapenem resistance differed between survivors and non-survivors in the unadjusted analyses, the direction of this association was not straightforward and did not parallel the strong association observed for MDR. Resistance categories are partly overlapping constructs, and their prognostic significance may depend on the microorganism involved, infection site, antimicrobial regimen, and patient severity. Therefore, the present findings support the use of MDR as the main resistance variable in the adjusted mortality model rather than interpreting each resistance phenotype in isolation.

### Strengths and Limitations

This study has several strengths. First, it integrates patient-level clinical characteristics, microbiological data, antimicrobial-resistance phenotypes, invasive procedures, and antimicrobial treatment within the same analysis. Second, mortality was evaluated using multivariable logistic regression rather than relying exclusively on bivariate associations. Third, patients with multiple infection episodes were analyzed at the patient level for the primary mortality outcome, thereby reducing the risk of treating repeated infection episodes as independent mortality observations.

Several limitations should also be acknowledged. The retrospective, single-center nature of the study limits causal inference and external generalizability. The exceptionally high proportion of mechanically ventilated patients indicates a highly selected case mix and limits direct comparison of the observed mortality with that reported in unselected HAI populations. The source analytic dataset did not include a standardized ICU-admission variable, infection-severity scores (APACHE II, SOFA, or SAPS), hepatic function, vasopressor/corticosteroid/immunosuppressive treatment, ICU length of stay before HAI, or a standardized history of previous hospitalizations. Polymicrobial infection was not encoded as a dedicated patient-level variable. These unavailable factors may have contributed to residual confounding. Although 192 patients and 242 infection episodes were included, only 31 patients survived, restricting the number of covariates that could be incorporated into multivariable models and contributing to wide confidence intervals. Complete-case selection before creation of the analytic extract cannot be fully reconstructed and may have introduced selection bias. Antimicrobial treatment was evaluated primarily by drug class, and treatment exposure could not be fully matched to isolate-level susceptibility to classify therapy as appropriate or inappropriate. The timing variable represented initiation of recorded post-culture treatment rather than time to the first microbiologically active agent. Treatment exposures accumulated during hospitalization are vulnerable to confounding by indication and time-dependent/immortal-time bias, because patients must survive long enough to receive later therapies. Finally, treatment duration is intrinsically vulnerable to survivorship bias and was therefore treated as descriptive only and not used to infer treatment benefit.

Overall, our findings highlight the prognostic relevance of multidrug resistance among patients with healthcare-associated infections. MDR phenotype remained independently associated with in-hospital mortality even after adjustment for relevant clinical characteristics, whereas associations between individual antimicrobial classes and survival were more complex and should be interpreted in the context of antimicrobial susceptibility and treatment selection. These results reinforce the importance of early microbiological diagnosis, local resistance surveillance, antimicrobial stewardship, and rapid access to active therapy for patients at risk of infection with multidrug-resistant organisms. Prospective studies incorporating standardized severity scores and isolate-level assessment of antimicrobial appropriateness are warranted to further clarify the interaction between antimicrobial resistance, treatment adequacy, and mortality.

## 4. Materials and Methods

### 4.1. Study Design and Setting

This retrospective observational cohort study included patients with confirmed HAIs hospitalized between 2020 and 2025 in multiple clinical departments of a hospital in Târgu-Mureș, Romania. The study population comprised 192 patients with a total of 242 documented HAI episodes. The analytic dataset supplied for the study contained the eligible records used for analysis; no additional patient records were excluded after creation of this analytic dataset. Among the 192 patients, 151 had one documented episode, 32 had two episodes, and 9 had three episodes. For mortality analyses, the patient represented the primary unit of analysis.

### 4.2. Study Population

Patients were eligible for inclusion if they had a healthcare-associated infection recorded in the national surveillance system according to the case definitions applicable under Romanian national surveillance regulations (Order of the Ministry of Health No. 1101/2016), which referred to the European case definitions in Commission Implementing Decision 2012/506/EU [20]. The present analysis used the eligible, anonymized records available in the analytic surveillance dataset. Because the study dataset was already assembled from reported HAI records, the number of records excluded before creation of the analytic extract could not be reconstructed; this is acknowledged as a potential source of selection bias.

### 4.3. Data Sources and Data Collection

Data were retrospectively extracted from the healthcare-associated infection surveillance database of the Mureș County Public Health Directorate. Information collected for each patient included demographic characteristics, hospital department, length of hospitalization, relevant comorbidities, previous antimicrobial exposure, invasive procedures, microbiological specimens, isolated microorganisms, antimicrobial susceptibility and resistance profiles, antimicrobial treatment, and in-hospital outcome. Comorbidities were derived from diagnoses recorded in the surveillance dataset and grouped into cardiovascular, pulmonary, oncological, metabolic, and renal categories. The available dataset documented that patients were transferred from various clinical and surgical departments to the ICU; however, it did not contain sufficiently detailed standardized information on the timing of ICU admission or ICU length of stay. Consequently, analyses according to ICU exposure duration or time from ICU admission to HAI onset could not be performed. Mechanical ventilation was retained as an available marker of critical illness and invasive supportive care.

### 4.4. Study Variables and Outcome Definition

The primary outcome was all-cause in-hospital mortality, defined as death occurring during the index hospitalization. Patients were consequently classified as non-survivors or survivors.

Demographic and clinical variables included age; sex; place of residence; cardiovascular, pulmonary, oncological, metabolic, and renal comorbidities; mechanical ventilation; central venous catheterization; recent surgery; previous antimicrobial therapy; and length of hospital stay.

Antimicrobial treatment variables included exposure to aminopenicillins, fluoroquinolones, aminoglycosides, glycylcycline antibiotics, oxazolidinones, cephalosporins, glycopeptides, carbapenems, and polymyxins. The index episode was defined as the first chronologically documented microbiological episode for each patient. In the primary patient-level treatment analysis, an antimicrobial-class exposure was coded as present if it was documented during any linked episode; this approach was retained to describe cumulative in-hospital antimicrobial exposure but may introduce time-dependent bias. A sensitivity analysis restricted antimicrobial exposures and MDR status to the index episode to assess the robustness of the principal findings.

### 4.5. Microbiological Assessment and Antimicrobial Resistance

Antimicrobial resistance phenotypes were categorized as multidrug-resistant (MDR), extensively drug-resistant (XDR), and pandrug-resistant (PDR) according to the standardized international definitions proposed by Magiorakos et al. [21]. MDR was defined as acquired non-susceptibility to at least one agent in three or more antimicrobial categories; XDR was defined as non-susceptibility to at least one agent in all but two or fewer antimicrobial categories (i.e., susceptibility retained to only one or two antimicrobial categories); and PDR was defined as non-susceptibility to all agents in all antimicrobial categories [21]. Carbapenem resistance (CR) was analyzed separately from the MDR/XDR/PDR classification.

For patients with multiple infection episodes, the primary patient-level analysis considered a resistance phenotype present if documented in at least one linked microbiological episode. For the graphical comparison of mortality across resistance categories, mutually exclusive groups were generated by assigning each patient to the highest resistance phenotype identified during hospitalization (PDR > XDR > MDR > non-MDR/XDR/PDR). Carbapenem resistance was analyzed as a separate phenotype rather than as an overlapping MDR indicator in the source coding. To address temporal-order concerns, a sensitivity analysis was additionally performed using resistance status from the index episode only.

### 4.6. Antimicrobial Treatment Assessment

Post-culture antimicrobial treatment was evaluated according to the antimicrobial classes administered after microbiological sampling. The index episode was defined as the first chronologically documented microbiological episode. Time to specific antimicrobial therapy was defined as the number of days between microbiological sampling and initiation of the recorded post-culture treatment for the index episode. The database did not reliably distinguish empirical from targeted treatment at the individual drug-isolate level, and susceptibility results were not linked to each administered drug in a manner that allowed for reproducible classification of therapy as microbiologically active or appropriate. Accordingly, treatment analyses are descriptive/exploratory and should not be interpreted as estimates of causal treatment efficacy.

Because microbiological susceptibility and administered treatment were not matched at the individual drug-isolate level for the present analysis, ‘specific antimicrobial therapy’ denotes recorded post-culture therapy and should not be interpreted as synonymous with ‘appropriate’, ‘active’, or susceptibility-guided therapy.

### 4.7. Statistical Analysis

Statistical analyses were performed at the patient level for the primary outcome of in-hospital mortality. Continuous variables were assessed for distribution and are presented as median and interquartile range (IQR) where appropriate, whereas categorical variables are reported as absolute frequencies and percentages. Continuous variables were compared between survivors and non-survivors using the Mann–Whitney U test. Categorical variables were compared using Pearson’s chi-square test or Fisher’s exact test, as appropriate according to expected cell frequencies.

Binary logistic regression was used to investigate factors associated with in-hospital mortality. Univariable logistic regression analyses were initially performed to estimate crude odds ratios (ORs) with 95% confidence intervals (CIs) for demographic and clinical characteristics, antimicrobial resistance phenotypes, and antimicrobial treatment variables.

Two deliberately parsimonious multivariable logistic regression models were constructed to limit overfitting given the small survivor group. Model 1 (clinical and antimicrobial-resistance model) included age, mechanical ventilation, recent surgery, and MDR phenotype. Model 2 (treatment-focused model) included age, mechanical ventilation, recent surgery, aminopenicillin exposure, second-generation aminoglycoside exposure, and third-generation cephalosporin exposure. These covariates were selected a priori on clinical grounds and to address the study hypotheses while limiting the number of parameters; they were not selected solely on the basis of univariable *p*-values. Adjusted odds ratios (aORs) with 95% CIs were reported.

Separate models were used because only 31 patients survived, which constrained the number of parameters that could be estimated reliably. Multicollinearity was assessed using variance inflation factors (VIFs); all VIFs were <1.12 in both models. No multiplicity correction was applied because the secondary comparisons were exploratory; consequently, nominal *p*-values should be interpreted cautiously in view of the number of comparisons. Variables used in the regression models had complete analyzable coding after application of the study’s predefined binary coding rules. Sensitivity analyses restricted MDR status and treatment exposure to the index episode.

All statistical tests were two-sided, and a *p*-value < 0.05 was considered statistically significant for the primary analyses. Secondary and treatment-related analyses were considered exploratory. Statistical analyses were performed using IBM SPSS Statistics for Windows, version 23 (IBM Corp., Armonk, NY, USA). Additional sensitivity and diagnostic analyses performed during revision reproduced the same patient-level coding and model specification.

## 5. Conclusions

In this retrospective cohort of patients with healthcare-associated infections, multidrug resistance was strongly associated with in-hospital mortality and remained independently associated with mortality after adjustment for relevant clinical factors. MDR phenotype was associated with substantially higher odds of death, underscoring the prognostic importance of antimicrobial resistance in hospitalized patients with HAIs.

Several antimicrobial treatment classes were associated with lower odds of mortality in exploratory observational analyses; however, these associations are hypothesis-generating only. They may reflect confounding by indication, differences in baseline severity and pathogen susceptibility, and time-dependent treatment exposure, and should not be interpreted as protective effects of specific antimicrobial classes.

The observed associations therefore do not establish a causal protective effect of specific antimicrobial classes.

No clear association was identified between the time to initiation of specific antimicrobial therapy and mortality, although this variable did not necessarily reflect time to microbiologically appropriate therapy.

These findings support the importance of antimicrobial resistance surveillance, rapid microbiological diagnosis, antimicrobial stewardship, and timely access to active treatment in patients at risk of MDR healthcare-associated infections. Future prospective studies incorporating standardized severity-of-illness scores and isolate-level assessment of treatment appropriateness are needed to better define the interaction between antimicrobial resistance, antimicrobial therapy, and mortality.

## Figures and Tables

**Table 1:** Patient characteristics according to in-hospital outcome.

Characteristic	Non-Survivors n = 161	Survivors n = 31	*p*-Value
Age, years, median (IQR)	71 (62–78)	62 (50.5–72)	*0.025*
Male sex, n (%)	101 (62.7%)	21 (67.7%)	0.596
Rural residence, n (%)	89 (55.3%)	19 (61.3%)	0.537
Cardiovascular comorbidities, n (%)	106 (65.8%)	19 (61.3%)	0.627
Pulmonary comorbidities, n (%)	35 (21.7%)	11 (35.5%)	0.101
Oncological comorbidities, n (%)	17 (10.6%)	4 (12.9%)	0.753
Metabolic comorbidities, n (%)	52 (32.3%)	10 (32.3%)	0.997
Renal comorbidities, n (%)	23 (14.3%)	2 (6.5%)	0.381
Mechanical ventilation, n (%)	148 (91.9%)	25 (80.6%)	0.092
Central venous catheter, n (%)	126 (78.3%)	26 (83.9%)	0.481
Previous antibiotic therapy, n (%)	152 (94.4%)	29 (93.5%)	0.693
Recent surgery, n (%)	28 (17.4%)	11 (35.5%)	*0.022*
Length of stay, days, median (IQR)	14 (9–20)	18 (7.5–33.5)	0.417

Continuous variables: Mann–Whitney U test. Categorical variables: Pearson χ^2^ or Fisher’s exact test, as appropriate.

**Table 2:** Contingency table of MDR and carbapenem-resistance coding at patient level.

Patient-Level Phenotype	CR Present	CR Absent	Total
MDR present	0	90	90
MDR absent	55	47	102
Total	55	137	192

**Table 3:** Microbiological characteristics and antimicrobial resistance according to in-hospital outcome.

Microbiological Characteristic	Non-Survivors n = 161	Survivors n = 31	*p*-Value
Antimicrobial resistance phenotypes			
MDR	83 (51.6%)	7 (22.6%)	0.003
XDR	32 (19.9%)	9 (29.0%)	0.255
Carbapenem-resistant phenotype	41 (25.5%)	14 (45.2%)	0.026
Selected microorganisms/resistance profiles			
*Acinetobacter baumannii MDR*	77 (47.8%)	4 (12.9%)	<0.001
*Acinetobacter baumannii CR*	10 (6.2%)	5 (16.1%)	0.072
*Acinetobacter baumannii*	57 (35.4%)	19 (61.3%)	0.007
*Pseudomonas aeruginosa*	17 (10.6%)	5 (16.1%)	0.363
*Pseudomonas aeruginosa CR*	4 (2.5%)	1 (3.2%)	0.590
*Klebsiella pneumoniae*	7 (4.3%)	2 (6.5%)	0.640
*Klebsiella pneumoniae CPE*	7 (4.3%)	4 (12.9%)	0.081

For patients with multiple infection episodes, microbiological variables were coded as present if documented in at least one linked episode (242 infections mapped to 192 patients).

**Table 4:** Post-culture antimicrobial treatment according to in-hospital outcome.

Antimicrobial Exposure/Treatment Characteristic	Non-Survivors n = 161	Survivors n = 31	*p*-Value
Aminopenicillins, n (%)	11 (6.8%)	10 (32.3%)	*<0.001*
2nd-generation fluoroquinolones, n (%)	31 (19.3%)	4 (12.9%)	0.402
3rd-generation fluoroquinolones, n (%)	11 (6.8%)	3 (9.7%)	0.704
2nd-generation aminoglycosides, n (%)	5 (3.1%)	7 (22.6%)	*<0.001*
3rd-generation aminoglycosides, n (%)	9 (5.6%)	5 (16.1%)	0.054
Glycylcyclines, n (%)	14 (8.7%)	1 (3.2%)	0.473
Oxazolidinones, n (%)	37 (23.0%)	4 (12.9%)	0.210
3rd-generation cephalosporins, n (%)	50 (31.1%)	17 (54.8%)	*0.011*
Glycopeptides, n (%)	34 (21.1%)	7 (22.6%)	0.856
Carbapenems, n (%)	85 (52.8%)	12 (38.7%)	0.151
Polymyxins, n (%)	62 (38.5%)	16 (51.6%)	0.174
Time to specific therapy, days, median (IQR)	6 (4–7)	6 (5–7)	0.541
Antibiotic treatment duration, days, median (IQR)	4 (2–8)	9 (5–19)	*<0.001*

Antimicrobial exposure was coded as any documented exposure across linked infection episodes. Time-to-treatment and duration refer to the index episode. Treatment-duration findings should be interpreted cautiously because of survivorship bias.

**Table 5:** Univariable logistic regression analysis of factors associated with in-hospital mortality.

Predictor	Crude OR	95% CI	*p*-Value
Age, per 1-year increase	1.03	1.00–1.06	0.031
Male sex	0.80	0.35–1.82	0.596
Mechanical ventilation	2.73	0.95–7.86	0.062
Central venous catheter	0.69	0.25–1.93	0.483
Recent surgery	0.38	0.17–0.89	0.025
MDR phenotype	3.65	1.49–8.95	0.005
XDR phenotype	0.61	0.25–1.44	0.258
Carbapenem-resistant phenotype	0.41	0.19–0.92	0.029
Aminopenicillins	0.15	0.06–0.41	<0.001
2nd-generation aminoglycosides	0.11	0.03–0.37	<0.001
3rd-generation aminoglycosides	0.31	0.10–0.99	0.048
3rd-generation cephalosporins	0.37	0.17–0.81	0.013
Carbapenems	1.77	0.81–3.89	0.154
Polymyxins	0.59	0.27–1.27	0.177

**Table 6:** Multivariable logistic regression models for in-hospital mortality.

Predictor	Model 1 aOR (95% CI)	*p*-Value	Model 2 aOR (95% CI)	*p*-Value
Age, per 1-year increase	1.03 (1.00–1.06)	0.067	1.03 (1.00–1.06)	0.097
Mechanical ventilation	2.79 (0.88–8.88)	0.082	2.63 (0.79–8.71)	0.114
Recent surgery	0.48 (0.20–1.19)	0.113	0.42 (0.15–1.15)	0.091
MDR phenotype	3.58 (1.42–8.98)	*0.007*	—	—
Aminopenicillins	—	—	0.21 (0.07–0.64)	*0.006*
2nd-generation aminoglycosides	—	—	0.23 (0.06–0.97)	*0.045*
3rd-generation cephalosporins	—	—	0.35 (0.14–0.88)	*0.025*

Model 1: clinical + antimicrobial-resistance model (age, mechanical ventilation, recent surgery, MDR). Model 2: treatment-focused model adjusted for age, mechanical ventilation, and recent surgery. aOR, adjusted odds ratio; CI, confidence interval.

## Data Availability

The data analyzed in this study were obtained from the healthcare-associated infection surveillance database of the Mureș County Public Health Directorate. Because the dataset contains sensitive health-surveillance information, it is not publicly available. Access to anonymized data may be considered by the data custodian upon reasonable request and subject to applicable institutional, legal, and data-protection requirements.
